# Description of a new quantitative method to assess mitochondrial distribution pattern in mature equine oocytes

**DOI:** 10.1007/s11259-024-10325-z

**Published:** 2024-02-10

**Authors:** Marcos Luis-Calero, Pablo Fernández-Hernández, José Manuel Ortiz-Rodríguez, Carmen Cristina Muñoz-García, Isaac Jardin, Beatriz Macías-García, Lauro González-Fernández

**Affiliations:** 1 Departamento de Medicina animal, Grupo de Investigación Medicina Interna Veterinaria (MINVET), Instituto de Investigación INBIO G+C, Universidad de Extremadura, Cáceres, Spain; 2 Department of Veterinary Medical Sciences (DIMEVET), University of Bologna, Bologna, Italy; 3 Departamento de Fisiología, Grupo de Investigación Fisiología Celular, Instituto Universitario de Biomarcadores de Patologías Metabólicas y Moleculares (IBPM), Universidad de Extremadura, Cáceres, Spain; 4 Departamento de Bioquímica y Biología Molecular y Genética, Grupo de Investigación Señalización Intracelular y Tecnología de la Reproducción (SINTREP), Instituto de Investigación INBIO G+C, Universidad de Extremadura, Av. de las Ciencias, s/n, Cáceres, 10004 Spain

**Keywords:** Mitochondrial distribution pattern, Oocyte, Cytoplasmic maturation, Quantitative assay, Equine

## Abstract

The Mitochondrial distribution pattern or MDP in mammalian oocytes serves as an indicator of their cytoplasmic maturity, with a heterogeneous pattern associated with mature cytoplasm. Currently, MDP assessment involves fluorescent labelling of mitochondria followed by visual evaluation, as no quantitative method exists. Our objective was to develop a quantitative approach to assess MDP in mature equine oocytes. Equine oocytes, obtained by ovum pick up (OPU) were matured in vitro, and only metaphase II oocytes were used in the study (*n* = 56). Following denudation, oocytes were fixed, stained with MitoTracker™ Red CMXRos (50 nM in TCM-199 with Hank´s salts and 10% FBS) for 15 min at 38 °C, and then incubated with 2.5 µg/ml Hoechst 33342 for 10 min at 38 °C. Confocal microscope images were acquired, and the oocyte’s MDP was visually classified as either homogeneous (HoD; *n* = 17) or heterogeneous (HeD; *n* = 39). For quantitative analysis, Fiji-ImageJ software was employed. Background subtraction was performed, and a 1-pixel line along the diameter was drawn to calculate the intensity profile. Fluorescence intensities were normalized, and ratios of peripheral to central fluorescence intensity were determined. Student´s t-test was used for comparations; MDP ratio was (mean ± standard error of the mean): 0.8 ± 0.02 for HoD and 0.3 ± 0.02 for HeD (*p* < 0.001). These results demonstrate concordance between quantitative and qualitative MDP assessment in mature equine oocytes. Our study describes a new approach to quantify mitochondrial distribution pattern and cytoplasmic maturation in mature equine oocytes.

## Introduction

Oocyte in vitro maturation (IVM) is routinely used for in vitro embryo production across various species (Hinrichs 2018). This process encompasses both nuclear and cytoplasmic maturation. Currently, commercial production of equine embryos in vitro involves the use of mature oocytes (Metaphase II) for intracytoplasmic sperm injection (ICSI), which is assessed by direct visualization of the first polar body extrusion. However, despite using mature oocytes, after ICSI only between 9.85 and 20.37% of them develop to the blastocyst stage (Lazzari et al. 2020), suggesting that equine oocytes may not acquire full cytoplasmatic maturation. Cytoplasmic maturation plays a crucial role in determining the oocyte´s developmental competence (Bavister and Squirrell 2000; Torner et al. 2007; Zhuang et al. 2012) and involves the redistribution of specific organelles and components, including mitochondria (Ferreira et al. 2009). During oocyte maturation, mitochondria increase in number and migrate within the oolemma. Evaluation of the mitochondrial distribution pattern (MDP) in mammalian oocytes is typically conducted through confocal microscopy after labelling mitochondria with specific fluorescent dyes (González et al. 2012; Liu et al. 2010; Torner et al. 2007; Zhuang et al. 2012). These MDPs exhibit variations between species, with clearly defined mitochondrial migration patterns in murine oocytes during cytokinesis (Kirillova et al. 2021). Currently, MDPs in equine oocytes are qualitatively assessed through visual image analysis, employing subjective criteria to categorize them as either homogeneous (indicating uniform mitochondrial distribution within the oolemma) associated with incomplete cytoplasmic maturation (Caillaud et al. 2005) or heterogeneous (clustered or aggregated), considered an indicator of cytoplasmic maturity of oocytes after IVM (Ambruosi et al. 2009; Torner et al. 2007). Despite mitochondrial activity is quantitatively measured by assessing overall fluorescence within the oolema (Clérico et al. 2021; Martino et al. 2014; Torner et al. 2007), a quantitative method for MDP evaluation is currently unavailable. Establishing a quantitative method for MDP evaluation in equine oocytes is crucial for researchers seeking to identify optimal in vitro conditions to obtain oocytes presenting a mature cytoplasm to improve in vitro embryo production. Therefore, our objective was to develop a quantitative method to assess mitochondrial distribution (homogeneous or heterogeneous) in equine oocytes matured in vitro.

## Materials and methods

### Oocyte source and in vitro maturation

Oocytes were retrieved by ovum pick up (OPU) following a previously described protocol (Fernández-Hernández et al. 2023) from 5 mares. Cumulus-oocyte complexes (COCs) were cultured in TCM-199 with Earle´s salts and 10% of Fetal Bovine Serum (FBS) (v/v; Gibco, Madrid Spain), 5 mU/ml of follicle-stimulating hormone (FSH), and 25 µg/ml of gentamycin in droplets at a rate of 20 µl/oocyte. The IVM droplets were covered with mineral oil (NidOil, Nidacon, Sweden), allowed to equilibrate for a minimum of 3 h, and IVM was conducted for 26–28 h at 38.2 °C under a 5% CO_2_ atmosphere in air with maximum humidity.

### Mitochondrial and chromatin staining

Following IVM, oocytes were denuded by gentle pipetting in PBS-PVA [PBS + 0.01% PVA (v/v)] with 0.1% hyaluronidase (w/v) in TCM-199 with Hank’s salts. Subsequently, oocytes were stained with MitoTracker™ Red CMXRos (Thermo Fisher Scientific, Madrid, Spain) at 50 nM in TCM-199 with Hank´s salts and 10% FBS (v/v) for 15 min at 38 °C in the dark. Afterwards, oocytes were fixed with 4% formaldehyde in PBS-PVA overnight at 4 °C in the dark. Fixed oocytes were washed in PBS-PVA and further incubated with 2.5 µg/ml Hoechst 33342 (Sigma-Aldrich, Barcelona, Spain) in PBS-PVA for 10 min at 38 °C in the dark. Stained oocytes were mounted on slides and evaluated.

### Evaluation of maturation status

Oocytes were classified based on their DNA conformation using a Nikon Eclipse 50i fluorescence microscope equipped with a mercury lamp and a 60X objective following previously validated criteria (González-Fernández et al. 2018). Only mature oocytes showing metaphase II chromatin configuration were used (*n* = 56).

### Confocal microscopy and mitochondrial distribution pattern evaluation

Fluorescence measurements were conducted on an inverted confocal microscope (Axio Observer 7, Carl Zeiss, Germany) using a LD LCI Plan-Apochromat 25×/0.8 multi-immersion objective at a zoom of 1.3× with image acquisition (Axiocam 712 mono, Carl Zeiss, Germany) and analysis system for videomicroscopy (ZEN Blue 3.4, Zeiss). Image processing and quantitative analyses were performed using Fiji-ImageJ software (NIH, Bethesda, MD, USA). Background was subtracted with the “subtract background” function (rolling ball radius = 250). Then qualitative and quantitative analysis of MDP were performed.

#### Qualitative analysis

**Fig. 1:**
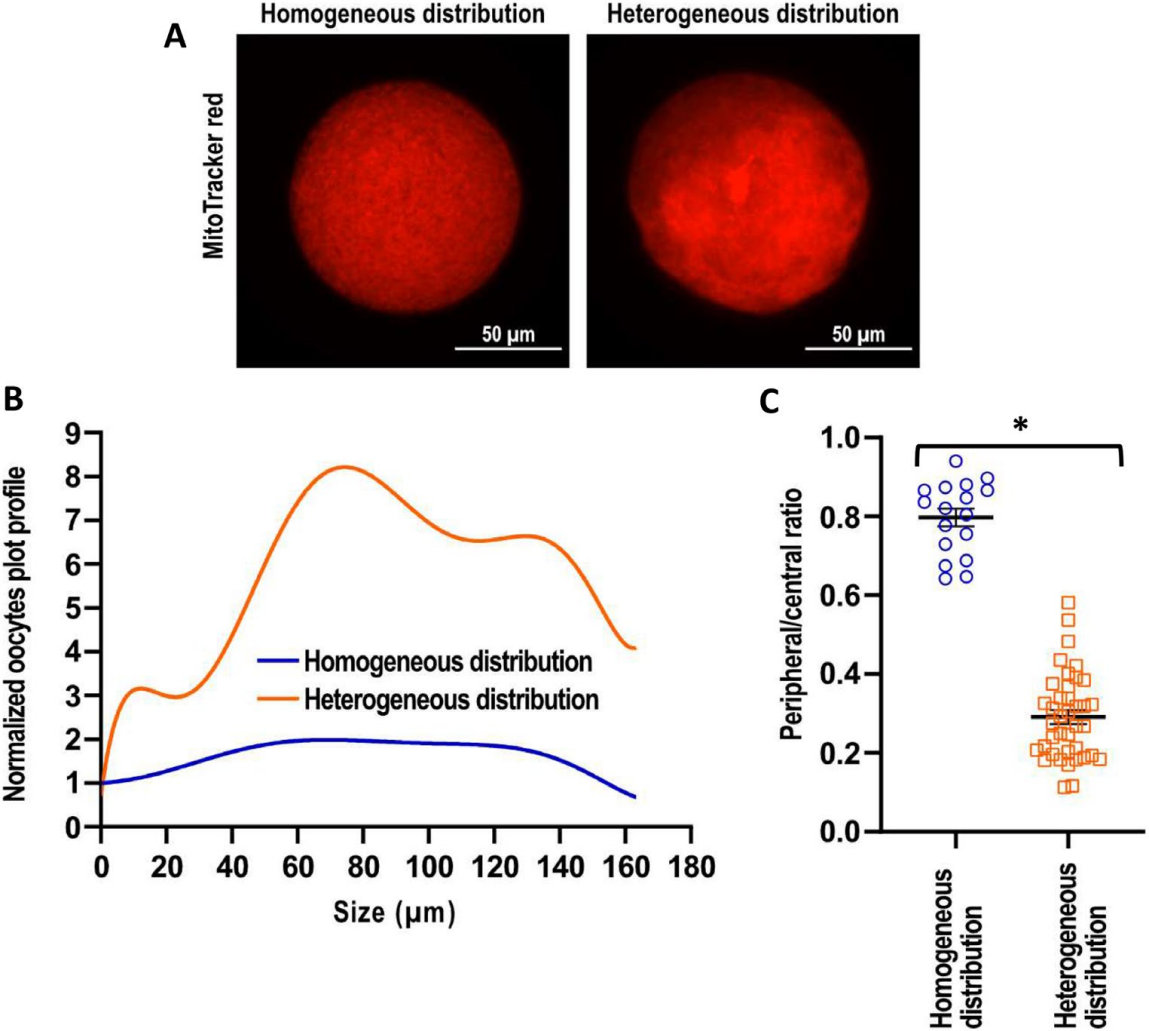
Assessment of mitochondrial distribution pattern (MDP) in mature equine oocytes using a qualitative **(A)** and a quantitative approach **(B, C)**. **(A)** Microphotographs of two oocytes stained with MitoTracker™ Red CMXRos showing homogeneous and heterogeneous MDP. **(B)** Normalized fitting curves showing profile intensities within the oocyte’s diameter for homogeneous (HoD; left picture) or heterogeneous (HeD; right picture) MDP. **(C)** Scatter plot showing the distribution of MDP ratio (peripheral/central fluorescence intensity) for individual HoD and HeD oocytes. Significant differences were observed between groups (*p* < 0.001)*

Oocytes were visually classified in two groups according to MPD following previously reported criteria (Ambruosi et al. 2009). Those showing an even mitochondrial distribution throughout the ooplasm were assigned to the homogeneous distribution (HoD) group, while those presenting aggregated or uneven fluorescence at the perinuclear region were classified as heterogenous (HeD; Fig. 1A).

#### Quantitative analysis

A 1-pixel line that spanned the diameter of the oocytes was manually drawn, and the intensity profile was calculated. Using FIJI’s “add fit” function, a fitting curve of the intensity profile was generated (Fig. 1B). Next, fluorescence intensities were normalized, and ratios of peripheral to central fluorescence intensity (MDP ratio) were calculated, giving a value of each oocyte’s ratio.

### Statistical analysis

Statistical analysis was performed using GraphPad Prism v.8.4.3 (GraphPad Software, San Diego, CA, USA). The gaussian distribution of the samples was assessed using a Shapiro-Wilk test, and then a t-test was used to compare MDP ratio between HoD and HeD groups (*p* < 0.05).

## Results

### Qualitative analysis

Homogeneous MDP was observed in 17 out of 56 evaluated oocytes (HoD, 31%), while the remaining 39 were considered to exhibit heterogeneous MDP (HeD, 69%).

### Quantitative analysis

MDP ratios, expressed as mean (arbitrary units) ± standard error of the mean, resulted in 0.8 ± 0.02 for HoD and 0.3 ± 0.02 for HeD. In HeD, normalized fitting curves showed increased fluorescence in the inner cytoplasm compared to the borders (Fig. 1B). Statistically significant differences were observed between groups (*p* < 0.001) (Fig. 1C).

## Discussion

Heterogeneous Distribution (HeD) in equine oocytes has been visually classified into different categories, including crystalline, granulated, or peripheral/perinuclear distribution (Ambruosi et al. 2009; Torner et al. 2007). In our study, equine oocytes at the MII stage were classified as either HoD or HeD based on the previously mentioned criteria. Among these, 69% were classified as having a heterogeneous distribution, aligning with previous reports in human and equine oocytes (Ambruosi et al. 2009; Liu et al. 2010; Torner et al. 2007). To design a quantitative method to assess MPD, we adapted a protocol from a previous publication (Sanchez-Collado et al. 2021). The adapted model was based on the translocation assay of Nuclear Factor of Activated T cells (NFAT), which is a transcription factor involved in breast cancer metastasis. NFAT once fluorescently labelled, translocate from the cytoplasm to the nuclear region, and using confocal microscopy, fluorescence intensity is measured for each nuclear and cytoplasmic area. Subsequently, regions of interest are delineated, and the ratio of nuclear to total NFAT is calculated on a cell-by-cell basis (Sanchez-Collado et al. 2021). However, NFAT is only circumscribed to the perinuclear region, while mitochondria in equine oocytes are distributed throughout the oolemma (Ambruosi et al. 2009; Torner et al. 2007), and for this reason the aforementioned approach could not be directly extrapolated. Therefore, a line was manually drawn crossing the diameter of the oocytes at the maximum fluorescence, and the intensity profile was calculated. This MDP ratio ranged from 0.1 (maximum difference between peripheral and central intensity) to 1 (uniform intensity). The MDP ratio was significantly lower in HeD oocytes compared to the HoD group (*p* < 0.001; Fig. 1C), with 0.6 identified as the limit between both mitochondrial conformations. These results demonstrate correspondence between quantitative and qualitative MDP in mature equine oocytes and provides a reliable species-specific tool for equine oocytes. Although more replicates are needed to establish a universal cut-off value, in view of our results, this limit may be easily determined.

To the best of our knowledge, this is the first quantitative method available to assess mitochondrial distribution pattern in mammalian oocytes. The MDP ratio could serve as a useful tool to quantitatively evaluate mitochondrial aggregation and cytoplasmic maturation in equine oocytes and other species, although the values may vary among species due to different oocyte size and/or mitochondrial distribution patterns.

## Data Availability

The data that support the findings of this study are available on request from the corresponding author.
